# The Causal Relationship Between Acne Vulgaris and BMI: A Mendelian Randomization Study

**DOI:** 10.1111/jocd.70092

**Published:** 2025-03-04

**Authors:** Zhifeng Li, Weikun Qi, Tianying Zang, Zhiyong Zhang

**Affiliations:** ^1^ Department of Comprehensive Plastic Surgery Plastic Surgery Hospital, Chinese Academy of Medical Sciences, Peking Union Medical College Beijing China; ^2^ Department of Maxillofacial Surgery, Plastic Surgery Hospital, Chinese Academy of Medical Sciences Peking Union Medical College Beijing China

**Keywords:** acne vulgaris, body mass index (BMI), inflammatory skin condition, Mendelian randomization

## Abstract

**Background:**

As an inflammatory skin condition, acne usually presents with a complex pathogenesis. Recent studies suggest that BMI may relate to the incidence of acne. Mendelian randomization is a statistical method that is used to evaluate the causal effects of exposure factors on outcome variables.

**Methods:**

We applied the inverse‐variance weighted (IVW) method to evaluate the causal effect as the primary analysis between BMI and acne in our two‐sample Mendelian randomization study. We included 58 SNPs accounting for 2.5% (R^2^) of the BMI variation as instrumental variables (IVs) for BMI‐acne causal estimations.

**Result:**

The F‐statistic obtained from the first stage of the MR regression model was 61. Importantly, the results from all three methods consistently indicated that an increase in BMI did not elevate the risk of acne, with each result reaching statistical significance. Cochran's Q test revealed no evidence of heterogeneity among the IV estimates for individual variants. Our I^2^ values suggested low heterogeneity, thereby reinforcing the reliability of the MR estimates. Additionally, the “leave‐one‐out” analysis confirmed that no single SNP disproportionately affected the IVW point estimate.

**Conclusion:**

Our findings suggested that there is no causal relationship between BMI and acne.

## 1 Introduction

As a globally prevalent disease, acne represents an inflammatory skin condition characterized by a complex pathogenesis. It is not merely a dermatological issue that affects extensive areas of the skin; rather, it significantly impacts an individual's appearance, demeanor, and sometimes imposes a severe psychological burden [1]. The global prevalence across all age groups is estimated at 9.38%, yet the incidence of acne varies widely among different countries and age demographics, with estimates indicating that between 35% and nearly 100% of adolescents experience acne at some stage [2]. Manifestations of acne, which can range from mild to severe, predominantly include pimples, papules, pustules, nodules, and cysts. Moreover, patients suffering from severe acne may encounter additional complications such as scarring, erythema, and hyperpigmentation, further exacerbating the overall impact of the condition.

Despite the high prevalence of acne, its exact pathophysiological mechanisms remain unclear. Acne is thought to be multifactorial, including genetic predispositions, hormonal disorders, abnormal follicular keratinization, skin colonization by *Propionibacterium acnes* , and disorders of sebum production [3]. Recent studies have also suggested that factors such as diet and weight may be associated with the incidence of acne, but the relationship in this regard has not yet been definitively concluded. BMI is usually used as a tool to evaluate an individual's healthiness, nutritional level, and obesity, and is calculated by dividing body weight by the square of height. Earlier Italian studies have found a significant association between BMI and the onset of acne, and a 2012 report noted that young Italian men affected by acne generally had higher BMI [4]. However, the sample size of this study was too small, and it was not possible to determine a causal relationship between acne and BMI. Another report noted that Italian adolescents and young adults with a lower BMI had a reduced risk of acne [5]. In contrast, an Israeli study found that overweight and obesity were negatively associated with acne among young people and that the relationship was dose dependent [6]. However, this study did not show a causal relationship, only a negative correlation. Another study from Taiwan also showed a negative correlation between BMI and the number of acne lesions in Taiwanese women with moderate‐to‐severe acne [7]. Unlike the above studies, a Poland study demonstrated no significant difference in the prevalence of acne among adolescents between the ages of 12 and 18 with different BMIs [8]. Based on the previous studies of the relationship of BMI and acne, it is not hard to find that though several studies have shown some association between BMI and acne, the precise causal relationship remains.

Mendelian randomization (MR) is a sophisticated statistical technique employed to evaluate the causal effects of exposure factors on outcome variables. Deriving its name from the pioneering geneticist Gregor Mendel, whose seminal research elucidated the randomness inherent in the process of meiosis, this method leverages the stochastic nature of genetic variation. By utilizing genetic variants as instrumental variables (IVs), MR capitalizes on this random assignment to estimate the causal impact of an exposure on an outcome. This approach is particularly advantageous as it mitigates the influence of potential confounders and counteracts the biases introduced by reverse causation, thereby allowing for robust causal inferences that align with stringent statistical assumptions [9].

The results of past studies are often contradictory, each possessing its own limitations. It remains unclear whether confounders exist between BMI and acne incidence, and whether a clear causal relationship between the two has been established. Consequently, MR studies are crucial for both preventing acne and elucidating its pathogenesis. To date, no MR studies have been conducted to evaluate the relationship between BMI and acne.

To fill this research gap, we performed a two‐sample MR analysis to explore whether there is a relationship between BMI and acne and, if so, exactly what the association is.

## 2 Materials and Methods

The foundational assumptions of MR analysis are as follows [1]. The IVs must have a strong association with the exposure factor(s), as evidenced by an F‐statistic > 10 [2]. The IVs should not influence the outcome directly, except through the exposure, verified through pleiotropy tests such as Mendelian Randomization Pleiotropy RESidual Sum and Outlier (MR‐PRESSO) [3]. The IVs must be independent of confounders, assessed using databases like PhenoScanner. This study utilized genome‐wide association study (GWAS) summary‐level data provided by the IEU OpenGWAS project, which is supported by the MRC Integrative Epidemiology Unit (IEU) at the University of Bristol. The project aggregated and analyzed GWAS data from sources such as the UK Biobank, published studies, and the FinnGen biobank. As this study relied on publicly available, anonymized, and de‐identified data, approval from the Ethical Review Authority was not required.

### 2.1 Data Source

The data for genetic variants associated with BMI, which served as the exposure variable in this study, were sourced from the UK Biobank, facilitated by the IEU OpenGWAS project. This project meticulously curated and analyzed genetic data to identify variants linked to BMI. On the other hand, the GWAS data related to acne were compiled from multiple sources, including the FinnGen biobank, the UK Biobank, and the study conducted by Teder‐Laving M et al. [10], all of which were accessed through the GWAS Catalog. These comprehensive datasets provide a robust foundation for understanding the genetic underpinnings of both BMI and acne. Detailed descriptions of the exposure and outcome datasets, including the methodologies and specific genetic variants involved, are presented in Table 1.

**TABLE 1:** Information on the exposures and outcome datasets.

Exposure or outcome	Data source (ID or author)	Population	Participants included in analysis	SNPs
Body mass index	UK Biobank by the IEU open GWAS project (ebi‐a‐GCST90013870)	European	407 609	10 783 680
Acne	FinnGen biobank by the IEU OpenGWAS project (finn‐b‐L12_ACNE)	European	1299 (cases)/211 139 (controls)	16 380 454
Acne	GWAS catalog (GCST90245818)	European	34 422 (cases)/364 991 (controls)	1 048 575
Acne	UK biobank (Data‐Field 20 002)	European	392 954	28 987 534

### 2.2 IV Selection

We initially identified 425 independent single‐nucleotide polymorphisms (SNPs) associated with BMI at a genome‐wide significance level (*p* < 5 × 10^−8^). These SNPs were then pruned for linkage disequilibrium (LD) to ensure independence (*r* ^2^ < 0.001; distance < 1000 kpb), which helps in minimizing the risk of confounding due to closely linked genetic variants. To further refine our selection, each SNP was screened for potential confounding factors and assessed to confirm that it did not influence the outcome through pathways unrelated to BMI. This screening was conducted using the PhenoScanner database (http://www.phenoscanner.medschl.cam.ac.uk/, accessed on March 30, 2024), a comprehensive resource for exploring gene–phenotype associations [11].

SNPs that showed significant associations with known risk factors for acne, such as androgen, estrogen, inflammation, insulin, and growth factors, were excluded if they met the Bonferroni‐corrected significance threshold (*p* < 9.9 × 10^−5^; 0.05/425) [12]. Additionally, SNPs that were strongly associated with the outcome (*p* < 5 × 10^−8^) were removed to avoid potential bias. We conducted a harmonization process to eliminate SNPs with palindromic sequences where the allele frequencies could introduce ambiguity, as well as those with inconsistent allele orientations between datasets.

Further filtering was applied to remove SNPs associated with other pleiotropic effects, such as those influencing physical inactivity, smoking, and alcohol consumption, based on previous studies [13, 14, 15]. To evaluate the strength of the remaining SNPs as IVs and avoid weak instrument bias, we calculated the *F*‐statistic using the formula *F* =$\frac{R^{2}}{1-R^{2}}$× $\frac{N-k-1}{k}$, where *N* represents the sample size and *k* the number of included SNPs. SNPs with an *F*‐statistic less than 10 were deemed weak instruments and subsequently excluded to ensure robust results [16].

After this rigorous selection process, we combined the remaining SNPs with the acne GWAS dataset, taking care to address any intermediate allele frequency issues among 17 palindromic SNPs. We also evaluated the potential pleiotropy of the selected SNPs using the MR‐PRESSO test [17] to detect and correct for horizontal pleiotropy. Ultimately, this thorough screening resulted in a final set of 58 SNPs that were considered valid IVs for our analysis, providing a solid foundation for investigating the genetic links between BMI and acne.

### 2.3 Statistical Analysis

#### 2.3.1 MR Analyses and Meta‐Analyses

In our two‐sample MR study, we employed the inverse‐variance weighted (IVW) method as the primary analysis to assess the causal relationship between BMI and acne. The IVW method estimates the causal effect by calculating the exposure–outcome effect for each SNP using the Wald Ratio method. It then conducts a weighted linear regression where the intercept is constrained to zero. This approach provides more accurate estimates and greater statistical power, assuming that the IVs meet the three core assumptions: relevance, independence, and exclusion restriction [18].

To validate the robustness of our findings, we compared the IVW results with those obtained from two additional MR methods: MR‐Egger and the weighted median method. The weighted median method is advantageous as it remains valid even if up to 50% of the IVs are invalid, allowing some flexibility in the presence of potential violations of MR assumptions. In contrast, the MR‐Egger method is more conservative and can produce unbiased estimates even if all IVs are potentially invalid, though it requires more stringent assumptions about the nature of pleiotropy among IVs. Consistent results across these three methods—IVW, MR‐Egger, and weighted median—would strengthen the evidence supporting a causal relationship between BMI and acne.

Furthermore, we conducted meta‐analyses using a fixed‐effects model to combine the results, accounting for any potential heterogeneity across studies. This comprehensive approach ensures that our conclusions are robust and reliable, providing a clearer understanding of the genetic links between BMI and acne.

#### 2.3.2 Heterogeneity and Sensitivity Analyses

To evaluate the heterogeneity within the IVW model, we used Cochran's Q test, with a p‐value of less than 0.05 indicating the presence of heterogeneity [19]. While heterogeneity suggests variability in the causal estimates from different SNPs, it does not necessarily invalidate the IVW model, as the model can still provide valid results in the presence of some heterogeneity. To identify potential directional pleiotropy—where SNPs affect the outcome through pathways other than the exposure of interest—we applied the MR‐Egger method, which allows for nonzero intercepts, indicating pleiotropic effects.

Furthermore, we conducted a leave‐one‐out analysis to assess the influence of individual SNPs on the overall causal estimate. This approach systematically removes each SNP, one at a time, to determine if the exclusion of any single SNP significantly alters the results, thereby identifying SNPs that may disproportionately influence the findings. To further refine our analysis, we used the MR‐PRESSO method to detect and remove outlier SNPs that could skew the results. After identifying and excluding these outliers, we repeated the MR analysis to confirm the robustness of our findings [20]. This comprehensive approach ensures that our results are not driven by pleiotropy or outliers, providing a more accurate estimate of the causal relationship between BMI and acne.

## 3 Results

### 3.1 Validity of IVs

We selected 58 SNPs as IVs to estimate the causal effect of BMI on acne. These SNPs collectively account for 2.5% (R^2^) of the variation in BMI, indicating that they have a reasonably strong association with the exposure variable. The F‐statistic derived from the first stage of the MR regression model was 44, which is well above the conventional threshold of 10, suggesting that our IVs are sufficiently strong and unlikely to introduce weak instrument bias. Detailed effect estimates for the associations between each SNP and both BMI and acne are provided in Supplementary Table S1, which includes the beta coefficients, standard errors, and p‐values for each SNP, offering a comprehensive overview of their contributions to the BMI–acne relationship.

### 3.2 MR and Meta‐Analyses

**FIGURE 1:**
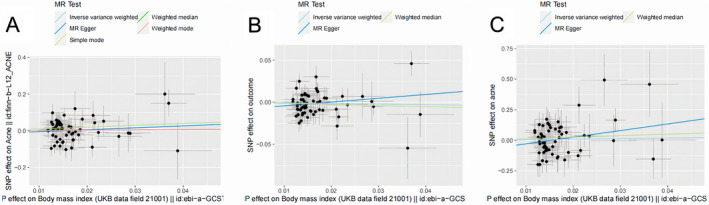
Scatter plots of genetic associations with BMI against the genetic associations with acne. The slopes of each line represent the causal association for each method. The light blue line represents the inverse‐variance weighted estimate, the dark blue line represents the MR‐Egger estimate, the light green line represents Simple mode estimate. (Figure A is the scatter plot of FinnGen biobank dataset, while B, C are the dataset of GWAS Catalog and UK biobank respectively).

**FIGURE 2:**
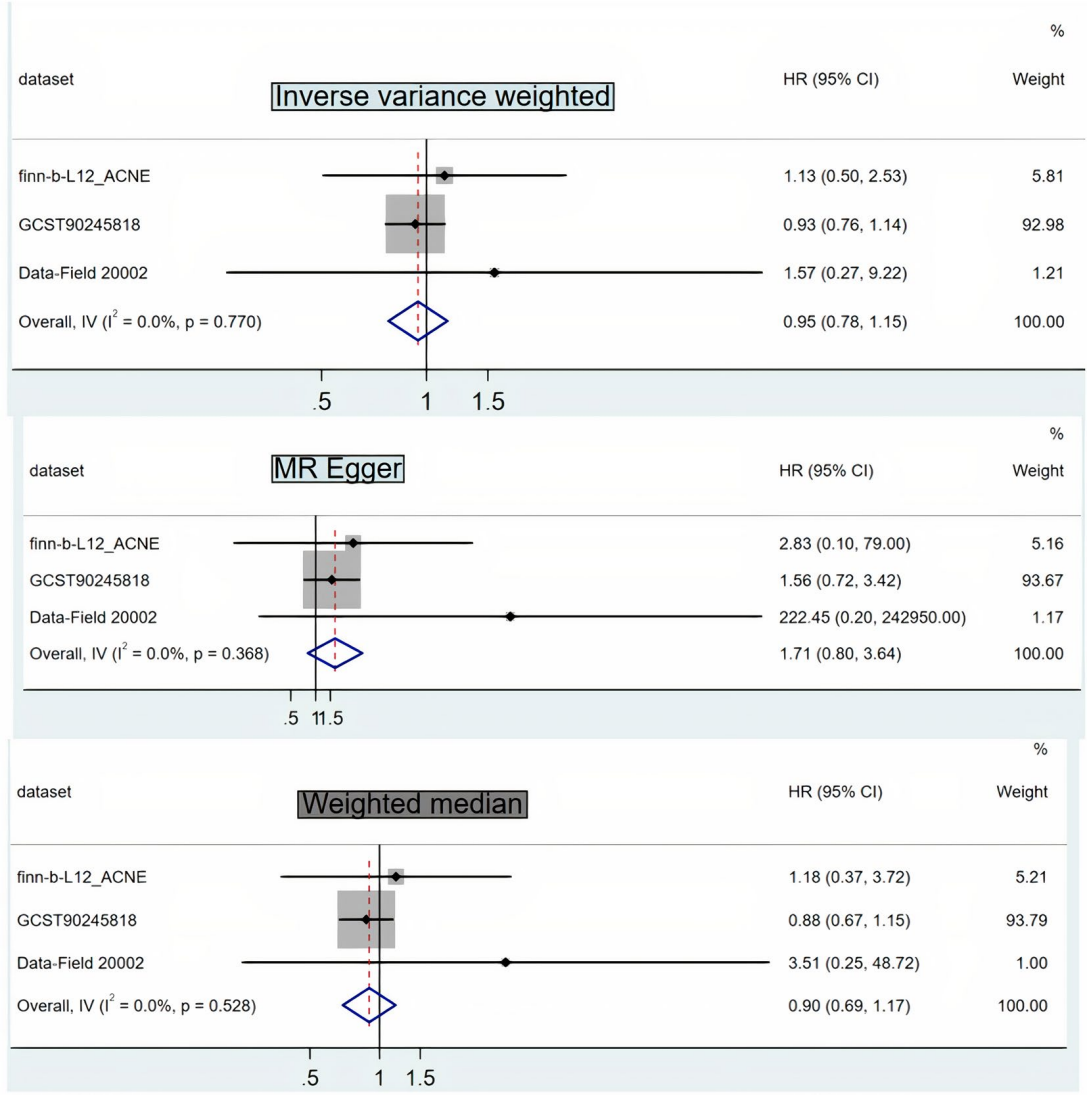
Forward MR analysis revealed no causal relationships between BMI and acne vulgaris risk. CI, confidence interval; IVW, inverse‐variance weighted; OR, odds ratio.

The results of three methods illustrated that the risk of acne did not increase with the increment of BMI for all three outcome datasets, and none of them achieved consistently statistical significance (Table 2 and Figure 1). Additionally, the meta‐analyses of the MR results of three datasets also proved that there is no relationship between BMI and acne (Figure 2).

**TABLE 2:** MR estimates from each method of assessing the causal effect of BMI on the risk of acne.

Data	MR method	No. of SNPs	Beta	SE	*p*	OR (95% CI)
FinnGen biobank by the IEU OpenGWAS project (finn‐b‐L12_ACNE)	IVW	58	0.119	0.412	0.773	1.126 (0.502–2.525)
	MR‐Egger	58	1.041	1.698	0.542	2.832 (0.102–79.001)
	Weighted median	58	0.192	0.177	0.279	1.212 (−0.155–0.539)
GWAS Catalog (GCST90245818)	IVW	58	−0.074	0.103	0.473	0.929 (0.759–1.137)
	MR‐Egger	58	0.448	0.399	0.266	1.565 (0.716–3.418)
	Weighted median	58	−0.133	0.138	0.338	0.876 (0.668–1.149)
UK biobank (Data‐field 20 002)	IVW	58	0.449	0.904	0.620	1.567 (0.266–9.221)
	MR‐Egger	58	5.405	3.569	0.136	222.449 (0.204–242949.958)
	Weighted median	58	1.256	1.342	0.349	3.511 (0.253–48.715)

Abbreviations: Beta, beta coefficient; IVW, inverse‐variance weighted; MR, Mendelian randomization; OR, odds ratio; SE, standard error; SNP, single‐nucleotide polymorphism.

### 3.3 Heterogeneity and Sensitivity Test

Cochran's Q test revealed no significant evidence of heterogeneity among the IV estimates derived from individual SNPs, as detailed in Table 3. Heterogeneity refers to the variability in causal estimates obtained from different SNPs, and a lack of heterogeneity suggests that the causal estimates are consistent across the SNPs used, thereby increasing the reliability of the MR results.

**TABLE 3:** The heterogeneity tests of the instrumental variables.

Data	MR method	Cochran's Q statistic	Heterogeneity *p*‐value
FinnGen biobank by the IEU OpenGWAS project (finn‐b‐L12_ACNE)	Inverse‐variance weighted	65.12	0.19
	MR‐Egger	65.48	0.21
GWAS catalog (GCST90245818)	Inverse‐variance weighted	77.25	0.038
	MR‐Egger	74.80	0.047
UK biobank (Data‐field 20 002)	Inverse‐variance weighted	64.33	0.24
	MR‐Egger	62.04	0.27

Additionally, our test for potential horizontal pleiotropy, which assesses whether SNPs influence the outcome through pathways other than the exposure of interest, indicated no significant violations, as shown in Table 4. This finding supports the validity of the IVs used in our analysis.

**TABLE 4:** The pleiotropy tests of the instrumental variables.

Data	MR method	Egger‐intercept	*p*‐value
FinnGen biobank by the IEU open GWAS project(finn‐b‐L12_ACNE)	MR Egger	‐0.02	0.58
GWAS Catalog (GCST90245818)	MR Egger	‐0.08	0.16
UK biobank (Data‐Field 20002)	MR Egger	‐0.01	0.18

**FIGURE 3:**
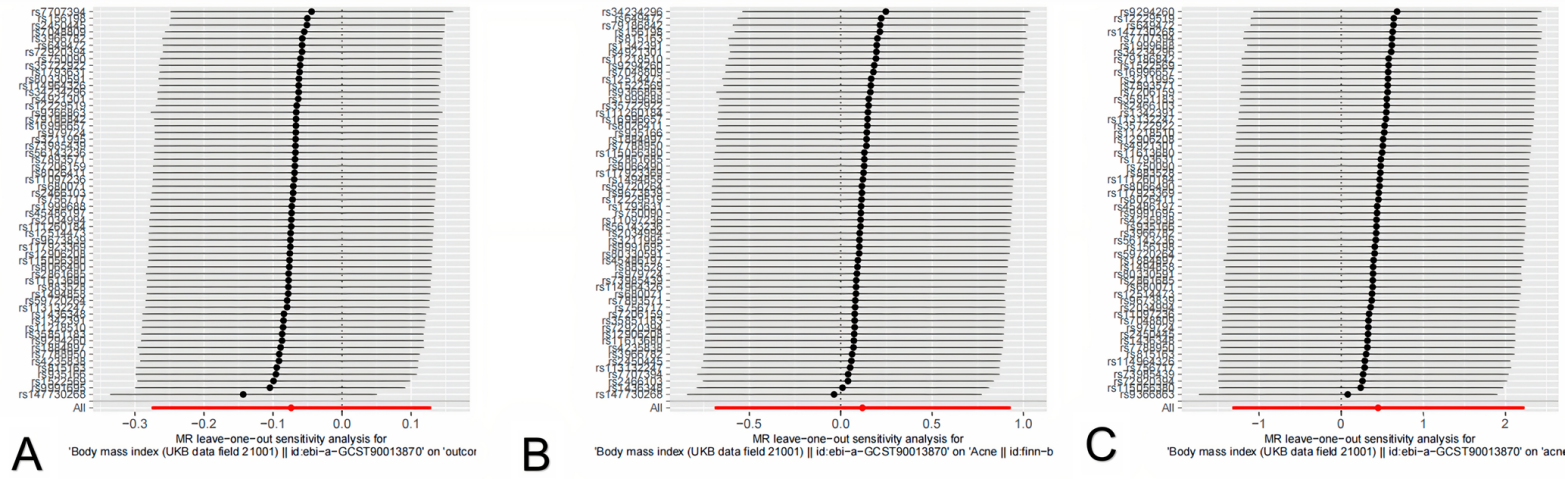
Leave‐one‐out analysis of the influence of each IV on the MR analysis. (Figure A is the scatter plot of FinnGen biobank dataset, while B, C are the dataset of GWAS Catalog and UK biobank respectively).

The results of the “leave‐one‐out” analysis further corroborated the robustness of our findings. This sensitivity analysis, illustrated in Figure 3, demonstrated that the overall IVW point estimate remained stable and was not significantly affected by the removal of any single SNP. This stability reinforces the reliability of our causal estimates and confirms that no individual SNP disproportionately influences the results.

## 4 Discussion

Acne, a prevalent global affliction, manifests through a spectrum of symptoms, spanning from mild pimples to severe nodules and cysts, often accompanied by scarring and pigmentation irregularities. Its worldwide incidence stands at approximately 9.38%, yet prevalence rates fluctuate significantly across demographics, with adolescents particularly susceptible, ranging from 35% to nearly 100%. The multifaceted pathogenesis of acne implicates various factors, including genetic predispositions, hormonal imbalances, aberrant follicular keratinization, *Acinetobacter* spp. colonization, and sebum production irregularities. Emerging research hints at a potential correlation between BMI and acne incidence, though conclusive evidence remains elusive, with recent investigations highlighting this association.

Previous studies are beset by several prominent issues. First, a pervasive lack of generalizability plagues much of the research landscape. Whether examining Lu et al.'s [7] focus on postadolescent females, Sas et al.'s [8] and Snast et al.'s [6] studies centered on adolescents, or Del Prete et al.'s [4] scrutiny of males, the majority of investigations are confined to specific regions or age cohorts, thus hampering their applicability to broader populations across diverse geographic and demographic spectra. Second, the recurrent reversal of conclusions exacerbates the quandary, exemplified by the conflicting findings of Del Prete et al. [4] and Di Landro et al. [5], suggesting a positive correlation between BMI and acne, juxtaposed with the contrasting outcomes of Snast and Lu et al. [6], which denote a negative correlation. The inherent error stemming from insufficiently robust population bases is readily apparent. Third, and perhaps most saliently, none of the aforementioned studies has succeeded in establishing a causal nexus between BMI and acne. Mere demonstration of correlation between the two variables falls short of furnishing the requisite rigorous evidence to underpin therapeutic interventions for acne sufferers or provide comprehensive life guidance.

This TSMR analysis demonstrated several key strengths. First, the MR method offered more reliable effect estimates compared to traditional observational studies by reducing the impact of confounders and minimizing the risk of reverse causality. This is achieved through the use of genetic variants as IVs, which are less susceptible to external biases. Second, the summary data used were from individuals of European descent, significantly mitigating the effects of population stratification and enhancing the accuracy of the findings. Lastly, the selection and identification of IVs followed a rigorous procedure, ensuring that only relevant and independent IVs were included. This thorough approach minimized the potential bias from unsuitable instruments, thereby reinforcing the validity and robustness of the causal estimates.

However, several limitations should be acknowledged. First, the data in this study were from individuals of European ancestry, leaving the situation for other ethnic groups unclear. Second, the lack of sex and age statistics hindered the possibility of conducting further subgroup analyses. Third, it may lead to different conclusions to adjust the BMI cutoff points in acne. However, the available data did not permit us to perform the MR analysis according to these adjusted BMI cutoff points.

While no causal relationship was found, it is hypothesized that genetic predispositions, metabolic pathways, BMI‐related factors (e.g., diet [21]), or inflammatory mediators may play a role in the observed lack of association. Future studies should investigate these aspects in more detail, possibly involving diverse populations and experimental designs.

Nevertheless, this meta‐analysis has its strengths. It is crucial that estimates of both the gene–exposure and gene–outcome associations are available for each of these variants. To the best of our knowledge, this is a rare study to investigate the causal relationship between BMI and acne.

## 5 Conclusion

In summary, our findings suggest that there is no statistically significant causal effect between BMI and acne.

In order to gain a deeper understanding of the complex relationship between BMI and acne, more population‐based surveys and experimental studies are needed in the future.

Although genetic evidence does not support this causal relationship, weight management remains important in the overall health management of acne patients, as it may still influence other metabolic or inflammatory pathways.

## Author Contributions

**Zhifeng Li:** conceptualization, data extraction and analysis, methodology, visualization. **Weikun Qi:** writing – original draft preparation. **Tianying Zang:** data extraction and analysis. **Zhiyong Zhang:** supervision.

## Ethics Statement

This study utilized publicly available, anonymized GWAS summary data. Ethical approval was waived per institutional guidelines, as no individual‐level data were accessed.

## Conflicts of Interest

The authors declare no conflicts of interest.

## Supporting information


Table S1.

## Data Availability

Data sharing not applicable to this article as no datasets were generated or analysed during the current study.
