# Supplementary material for: The Causal Relationship Between Acne Vulgaris and BMI: A Mendelian Randomization Study
Source: J Cosmet Dermatol. 2025 Mar 4;24(3):e70092. doi: 10.1111/jocd.70092 (PMC11877415; doi:10.1111/jocd.70092)
Supplement: Supplementary file 1 — Table S1. [file JOCD-24-e70092-s001.docx]

Supplementary table s1
